# Whole Genome Sequencing Investigation of a Tuberculosis Outbreak in Port-au-Prince, Haiti Caused by a Strain with a “Low-Level” *rpoB* Mutation L511P – Insights into a Mechanism of Resistance Escalation

**DOI:** 10.1371/journal.pone.0129207

**Published:** 2015-06-03

**Authors:** Oksana Ocheretina, Lishuang Shen, Vincent E. Escuyer, Marie-Marcelle Mabou, Gertrude Royal-Mardi, Sean E. Collins, Jean W. Pape, Daniel W. Fitzgerald

**Affiliations:** 1 Center for Global Health, Division of Infectious Diseases, Department of Medicine, Weill Cornell Medical College, New York, New York, United States of America; 2 Les Centres GHESKIO, Port-au-Prince, Haiti; 3 Biomedical Sciences (VTBMC), Cornell University, Ithaca, New York, United States of America; 4 Laboratory of Mycobacteriology, Wadsworth Center, New York State Department of Health, Albany, New York, United States of America; St. Petersburg Pasteur Institute, RUSSIAN FEDERATION

## Abstract

The World Health Organization recommends diagnosing Multidrug-Resistant Tuberculosis (MDR-TB) in high burden countries by detection of mutations in Rifampin (RIF) Resistance Determining Region of *Mycobacterium tuberculosis rpoB* gene with rapid molecular tests GeneXpert MTB/RIF and Hain MTBDR*plus*. Such mutations are found in >95% of *Mycobacterium tuberculosis* strains resistant to RIF by conventional culture-based drug susceptibility testing (DST). However routine diagnostic screening with molecular tests uncovered specific “low level” *rpoB* mutations conferring resistance to RIF below the critical concentration of 1 μg/ml in some phenotypically susceptible strains. Cases with discrepant phenotypic (susceptible) and genotypic (resistant) results for resistance to RIF account for at least 10% of resistant diagnoses by molecular tests and urgently require new guidelines to inform therapeutic decision making. Eight strains with a “low level” *rpoB* mutation L511P were isolated by GHESKIO laboratory between 2008 and 2012 from 6 HIV-negative and 2 HIV-positive patients during routine molecular testing. Five isolates with a single L511P mutation and two isolates with double mutation L511P&M515T had MICs for RIF between 0.125 and 0.5 μg/ml and tested susceptible in culture-based DST. The eighth isolate carried a double mutation L511P&D516C and was phenotypically resistant to RIF. All eight strains shared the same spoligotype SIT 53 commonly found in Haiti but classic epidemiological investigation failed to uncover direct contacts between the patients. Whole Genome Sequencing (WGS) revealed that L511P cluster isolates resulted from a clonal expansion of an ancestral strain resistant to Isoniazid and to a very low level of RIF. Under the selective pressure of RIF-based therapy the strain acquired mutation in the M306 codon of *embB* followed by secondary mutations in *rpoB* and escalation of resistance level. This scenario highlights the importance of subcritical resistance to RIF for both clinical management of patients and public health and provides support for introducing *rpoB* mutations as proxy for MICs into laboratory diagnosis of RIF resistance. This study illustrates that WGS is a promising multi-purpose genotyping tool for high-burden settings as it provides both “gold standard” sequencing results for prediction of drug susceptibility and a high-resolution data for epidemiological investigation in a single assay.

## Introduction

Multidrug resistant tuberculosis (MDR-TB) is defined by resistance to at least two anti-tuberculosis drugs—Rifampin (RIF) and Isoniazid (INH). MDR-TB cases do not respond to the standard therapy and require prolonged and expensive treatment with toxic second-line antibiotics [1]. Resistance to RIF is frequently accompanied by resistance to INH and is used as a surrogate marker for MDR-TB [2,3]. Furthermore, the World Health Organization (WHO) recommends treating patients infected with RIF mono-resistant strains with same second-line antibiotics as MDR-TB patients [4]. Therefore rapid and accurate laboratory diagnosis of resistance to RIF is a key factor for selecting appropriate treatment regimen and limiting transmission of drug resistant disease.

Traditional culture-based Drug Susceptibility Testing (DST) is based on monitoring growth of *Mycobacterium tuberculosis* (MTB) in medium supplemented with a critical concentration of RIF, currently set at 1 μg/ml [5]. Due to the slow growth rate of the organism, turn around time for laboratory diagnosis of RIF resistance lies between one and two months. The past several years have seen a change in the paradigm of testing for RIF resistance with the increased use of rapid molecular tests based on the detection of mutations in the 81 bp Rifampin Resistance Determining Region [RRDR] of the *rpoB* gene. Such mutations are found in >95% of MTB strains resistant to RIF by culture-based DST [6,7]. Because molecular tests PCR-amplify bacterial DNA, they can be utilized directly on clinical samples to diagnose resistance to RIF within days (MDRTBplus) or hours (GeneXpert MTB/RIF).

Numerous studies demonstrated high sensitivity of molecular tests for early detection of phenotypically RIF-resistant cases [8–10]. However their use for routine diagnostic screening uncovered unexpected *rpoB* mutations in some MTB strains phenotypically susceptible to RIF. Cases with discrepant genotypic (resistant) and phenotypic (susceptible) RIF susceptibility results account for at least 10% of all RIF-resistant diagnoses obtained with the molecular tests [11–14] and present a novel challenge for interpretation of laboratory findings. Some of the discrepant strains harbor “low-level” *rpoB* mutations that confer MICs above the background level determined for fully susceptible strains but below the critical concentration of 1 μg/ml currently used in standardized culture-based susceptibility tests [14–16].

There is an increasing evidence that the subcritical level of resistance to RIF can negatively impact clinical outcomes of treatment with standard RIF-based TB regimens [11, 17]. However the scope of the problem it poses for public health remains unknown. Isolates with *rpoB* mutations causing a low level resistance to RIF were reported to be rare in clinical practice and difficult to propagate *in vivo* [6, 11, 15]. They were mostly documented in treatment failure and relapse cases [11] or in immune-compromised patients [18]. These findings supported the hypothesis that “low-level” *rpoB* mutations impose a high fitness cost on MTB strains, which limits their infectivity and transmissibility [19–22].

The Mycobacteriology Laboratory at the Groupe Haïtien d'Etude du Sarcome de Kaposi et des Infections Opportunistes [GHESKIO] in Port-au-Prince, Haiti, detected several types of “low level” *rpoB* mutations in clinical MTB isolates with discrepant phenotypic and genotypic susceptibility results during routine screening of diagnostic samples with molecular tests [14]. Strains with a particular low-level resistance mutation obtained from independent patients were often clustered by spoligotyping, which suggested that they might be circulating in the community. We applied Whole Genome Sequencing (WGS) to investigate one suspected outbreak of 8 cases with a “low level” *rpoB* mutation L511P.

## Materials and Methods

### Ethics statement

The study was approved by the Institutional Review Board of Weill Cornell Medical College (New York, USA) and the Institutional Review Board of GHESKIO Centres (Port-au-Prince, Haiti). Clinical and epidemiological data were extracted from patients’ charts. As this was a retrospective clinical chart review, the requirement for informed consent was waived by the institutional review boards.

### Laboratory procedures

Primary specimens and MTB isolates in GHESKIO Mycobacteriology laboratory were routinely screened for mutations conferring resistance to RIF with MTBDR*plus* (Hain Life Sciences, Nehren, Germany) or/and GeneXpert MTB/RIF (Cepheid, CA, USA) assays. Isolates found resistant with molecular tests were further analyzed by sequencing of genes linked to resistance to RIF (*rpoB*), INH (*katG*, *inhA*, *ahpC*), ethambutol (EMB) (*embB*), pyrazinamide (PZA) (*pncA*) and fluoroquinolones (*gyrA*) and by spoligotyping. Isolates were also tested with conventional phenotypic DST on solid and liquid media. Minimal Inhibitory Concentration (MIC) to RIF was determined with Alamar Blue Broth Microdilution assay in 96-well plates. Laboratory procedures were described in detail elsewhere [14].

### Mycobacterial isolates

Eight MTB isolates from the previous report [14] were found to have *rpoB* mutation L511P and were further investigated in this study. Patients’ IDs correspond to the strain IDs used in a previous report as following: A—2010–50; B—2011–87; C—2011–93; D—2011–102; E—2011–76; F—2010–68; G—2008–26; H—2008–18; outgroup—2010–2.

### TB treatment in Haiti

Patients are treated using Directly Observed Therapy according to the WHO guidelines [23–26]. Category I regimen consists of four drugs RIF, INH, EMB and PZA. Patients with drug-susceptible TB with recurrence, treatment failure or default receive category II treatment, which includes the addition of streptomycin. Those with MDR-TB receive an individualized WHO category IV regimen including at least 4 active drugs and one injectable agent (kanamycin or capreomycin) based on the DST results.

### Classic epidemiological investigation

Clinical data were retrospectively extracted from patient’s charts including epidemiological questionnaires administered to all individuals treated in GHESKIO’s MDR-TB treatment hospital. The questionnaires were adapted for use in Haiti from Gardy et al. [27]. Notification and testing of close contacts was done with the consent of the patient.

### Whole Genome Sequencing and data analysis

Frozen primary MGIT cultures were re-grown on Lowenstein Jensen slant, DNA was extracted with CTAB method [28] and additionally purified with Qiagen DNeasy Blood & Tissue Kit [QIAGEN, Hilden, Germany]. One microgram of purified DNA was used to prepare DNA insert libraries of 150–250 bp with Illumina Genomic sample kit (Illumina, Inc, San Diego, CA). Sequencing was performed on Illumina HiSeq 2000 analyzer according to the manufacturer’s instructions. Single-end 50 bp reads were aligned to MTB reference genome H37Rv (GenBank NC_000962.2) with Novalign package (Novocraft Inc.; v.2.0.7). Single Nucleotide Polymorphisms (SNPs) and short INDEL variant discovery, genotyping and filtering were carried out with the Genome Analysis Toolkit version 1.6–6 [29]. SNP, INDEL discovery and genotyping were done on each sample and on all 7 samples simultaneously using standard hard filtering parameters or variant quality score recalibration [30].

Short reads were assembled into contigs with velvet (v.1.2.03) using kmer size 41 after comparing results using kmer sizes 27 to 47. Total assembled contigs lengths amounted to 98% of the reference genome. The contigs were mapped to the reference genome with BLAT and BLAST. SNP positions in highly similar, paralog gene families (PPE, PE_PGRS and wag22) and those where one or more isolates displayed an ambiguous residue with over 20% match with reference alleles were excluded. Mutation effects on protein and RNA genes were determined with Variant Effect Predictor [31] based on H37Rv annotations. All sequence data used in this work have been deposited into SRA under accession number “experiment SRX750472”.

23 primer pairs for confirmatory Sanger sequencing (3730XL) were designed with BLAST using 1000 bp upstream and downstream of the SNP. All sequencing was performed in Cornell University Bio Resource Center, Ithaca, NY.

## Results

### 1. L511P MTB cluster

From March 2008 to June 2012 153 individual MTB isolates with mutations in *rpoB* RRDR were found by routine screening of clinical specimens and isolates in GHESKIO TB laboratory in Port-au-Prince, Haiti [14]. Eight of them (5.2%) harbored a “low level” *rpoB* mutation L511P (cTg->cCg). Five isolates with a single L511P mutation and two isolates with double mutation L511P&M515T had MICs to RIF between 0.125 and 0.5 μg/ml and tested susceptible to RIF in culture-based DST. The eighth isolate with a double mutation L511P&D516C was phenotypically RIF-resistant (Table 1). All eight strains shared the same spoligotype SIT 53, and were tentatively designated as an L511P cluster.

**Table 1:** Clinical and microbiological characteristics of 8 patients in L511P cluster.

Patient	Age	Sex	HIV status	Category I treatment outcome	Category II treatment outcome	Category IV treatment outcome	*rpoB mutations*	Resistance to 1 μg/ml RIF (MGIT)	RIF MIC (μg/ml)
**A**	28	f	N	failed	failed	cured	L511P, D516C	RESISTANT	>8
**B**	34	f	N	failed	failed	cured	L511P, M515T	susceptible	[0.25–0.5]
**C**	10 m	m	N	…	…	cured	L511P, M515T	susceptible	[0.25–0.5]
**D**	30	m	P	failed	cured*	…	L511P	susceptible	0.125
**E**	28	f	P	cured	…	…	L511P	susceptible	[0.125–0.25]
**F**	21	f	N	…	…	cured	L511P	susceptible	0.125
**G**	18	m	N	failed**	…	…	L511P	susceptible	n.d.
**H**	30	f	N	unknown	unknown	…	L511P	susceptible	n.d.

* Delayed response

** Deceased

n.d. Not determined

### 2. Patients—clinical history and epidemiological investigation

**Fig 1:**
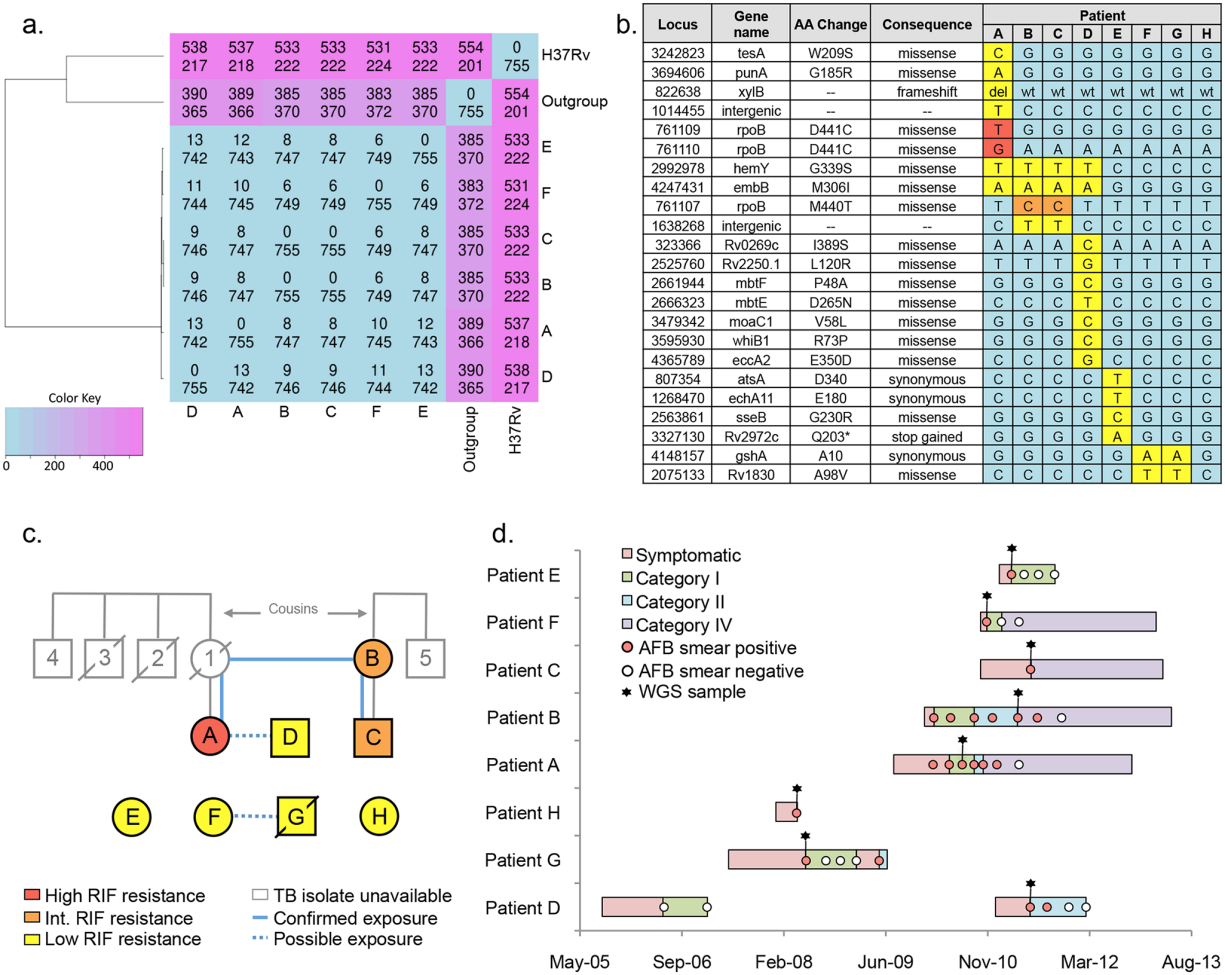
Investigation of the L511P cluster. (a) Number of SNPs different (above) and common (below) between any two isolates. H37Rv—reference genome, “outgroup”—unrelated clinical isolate with same spoligotype (b) Matrix of unique nucleotide variants found in L511P cluster. Secondary *rpoB* mutations responsible for escalation of RIF resistance level are in orange (intermediate level) and red (high level). M440T and D441C correspond to M515T and D516C in traditional *E*.*coli* numbering of *rpoB* codons. Isolates from patients G and H were not available for WGS and only positions presented in the matrix were interrogated by Sanger sequencing (c) Links between patients in L511P cluster established with interview. Confirmed and possible contacts between patients are shown in blue. Family members with history of TB and family ties are shown in grey. Crossed out shapes indicate patients who died of TB. (d) Timelines of treatment from onset of symptoms to completion of therapy. Negative AFB smears performed for monitoring of Category IV treatment have been omitted.

Clinical and epidemiological characteristics of the 8 patients from L511P cluster are presented in Table 1 and Fig 1d. Remarkably none of the interviewed patients reported contact with any other patient in the group, however we identified a few possible cases of indirect exposure.

Patient A was referred to GHESKIO center in 2010 after failing Category I and Category II TB treatments. Her first cousin once removed patient B independently entered MDR-TB treatment with a 10-month old son (patient C) in 2011. Both women belonged to the same extended family with significant history of pulmonary tuberculosis and treatment failure (Fig 1c). However they lived in different cities 50 miles apart and never knowingly met before admittance to GHESKIO MDR-TB hospital. Patient A’s mother was diagnosed with TB in 1996. Patient A lived with and took care of her mother until the latter died in 1997. Their house was frequented by extended family. Subsequently four close family members developed TB. Two of them, who were HIV co-infected, but not on the antiretroviral therapy (ART), died while receiving Category I TB treatment in 1998 and 1999. Two others, who were HIV-negative, were successfully cured with Category I treatment in 2007 and 2009. Patient B reported seeing patient A’s mother when she had active TB in 1996.

HIV co-infected patient D was treated for TB with Category I regimen in different health care facilities in 2005 and 2006. In 2010 he had recurrent TB after discontinuing ART, was treated with Category I regimen in GHESKIO, failed and was switched to Category II regimen. He showed delayed response to Category II treatment with AFB smear staying positive after 3 months. After 5 months his symptoms improved, AFB smear converted to negative and he is currently remaining symptom-free. This patient lived in the same densely populated poor neighborhood of Port-au-Prince as patient A but did not knowingly have social interaction with her or her extended family.

Patient G was treated for TB with Category I regimen in 2008 and was declared cured. However shortly after in 2009 he relapsed and died before his drug regimen could be adjusted. Patients E and F presented with their first episodes of TB in 2010 and 2011 respectfully. Patient E was cured with Category I treatment, Patient F was placed on Category IV treatment following a RIF-resistant result of MTBDR*plus* assay and was also cured. Of note, patient F reported living in the same neighborhood as patient G back in 2008 when he was having TB symptoms. The eighth patient H, who was diagnosed in 2008 was lost to follow-up and her epidemiological data were unavailable.

### 3. Whole Genome Sequencing of the L511P MTB cluster isolates

While epidemiological investigation uncovered very few links between the 8 cases with *rpoB* mutation L511P, common spoligotype SIT 53 suggested that they might belong to an outbreak. Spoligotyping separates MTB isolates into “types” according to presence or absence of 43 spacers in the Direct Repeat locus. However this genotyping method is not sufficiently discriminative to demonstrate that the isolates are closely related, especially since SIT 53 is common in Haiti and accounts for 6–7% of circulating MTB strains [32].

Therefore we subjected MTB isolates from 6 patients—A, B, C, D, E and F to Whole Genome Sequencing (WGS) to test the hypothesis that the L511P cluster was an outbreak and to try to delineate its evolutionary history. MTB isolates obtained from patients G and H couldn’t be analyzed because they were not viable in 2012.

An unrelated MDR-TB isolate with the same spoligotype SIT 53 but without the L511P *rpoB* mutation was also selected for WGS. This isolate is referred to as “outgroup” in the text and Fig 1.

Alignment of sequencing reads to the 4.41 Mb of the reference MTB genome H37Rv confirmed the SIT 53 spoligotype pattern in all 7 isolates and identified a total of 755 positions that had SNPs, short insertions or deletions in at least one of them (Fig 1a). The 6 isolates from the suspected outbreak were separated from the reference strain H37Rv by 527–531 sequence variants. They were closer to the Haitian outgroup isolate with the same spoligotype SIT 53 with distances between 383 and 390 sequence variants.

At the same time only 22 “non-identical SNPs” and 1 short deletion of 4 nucleotides were identified between the 6 L511P cluster isolates, all of which were subsequently confirmed by Sanger sequencing (Fig 1B). Individual members of the cluster demonstrated two (F) four (B, C and E), eight (A) and nine (D) non-identical sequence variants. These results confirmed that TB infection of the 6 patients resulted from the clonal expansion of a single ancestral strain and investigated cluster of cases presented an outbreak.

Isolates from the mother B and her baby C appeared identical. Both had 4 SNPs and shared 2 of them with patient A (a family member) and patient D (a possible neighbor of A). Therefore WGS confirmed the putative links established by the classic epidemiological investigation and indicated that patients A, B, C and D shared a more recent common ancestor.

Conserved DNA extracts from 2 isolates unavailable for WGS were amplified with primer pairs used to confirm the L511P cluster’s 23 non-identical sequence variants and analyzed with Sanger sequencing (Fig 1B). We discovered that patient G’s isolate had SNP profile identical with the isolate from patient F. Therefore WGS established a novel connection between the two patients, not previously identified by the classic epidemiological investigation.

### 4. SNPs in the L511P MTB cluster associated with drug resistance

Twenty three unique and 528 shared sequence variants found in the 6 outbreak isolates were examined for known links to resistance to anti-tuberculosis drugs (S1 Table), [33–35]. Variants found in the outgroup isolate are not discussed here.

WGS confirmed mutations in katG (S315T, all 6 strains), embB (M306I, strains A, B, C and D) and rpoB (L511P in all 6 strains, D516C in strain A, M515T in strains B and C) identified with Sanger sequencing. We found no mutations in *rpoA* or *rpoC* genes known to compensate for the fitness cost in RIF-resistant strains [34–36].

Mutations in gyrA (E21Q) and gyrB (K526Q) were found in all sequenced strains. However they were not associated with resistance to Fluoroquinolones as strains tested susceptible to Ofloxacin in a concentration range of 1 to 0.031 μg/ml. Full list of annotated sequence variants is available upon request.

## Discussion

Use of molecular tools improves the accuracy and resolution of classic epidemiological investigations [37], which is especially needed in high-burden settings, where transmission often occurs outside of the household through social interactions untraceable by traditional methods [38–40]. In particular this applies to Haiti where 1.5 million people in densely populated Port-au-Prince metropolitan area were displaced into tent camps after the earthquake of January 2010 [41]. Traditional MTB genotyping methods—IS*6110* RFLP, Spoligotyping and MIRU-VNTR each capture changes in only small part of MTB genome and are often used in tandem combinations [42, 43]. As cost of WGS is rapidly declining, it is increasingly utilized to investigate outbreaks [27, 44, 45], to predict resistance to drugs [35] and to delineate evolution of MDR-TB strains [37]. Arguably, WGS could be a powerful tool in countries like Haiti where the capacity for genotyping is not adequate or altogether non-existent.

Our results demonstrated superior resolution power of the WGS typing in high prevalence setting when compared to classic epidemiological investigation—it was able to confirm a possible indirect link between patients (A and B) and to uncover unknown links (D with A and B; F with G). At least four patients with history of treatment failure would likely have been misclassified as cases with acquired resistance, while WGS unequivocally demonstrated that they were cases of primary MDR-TB. Our unpublished data indicate that primary MDR-TB is an important factor driving MDR-TB epidemics in Haiti and so resources need to be allocated not only for MDR-TB diagnostics and treatment but also for the active tracing and screening of contacts of known TB patients. The eight isolates characterized in this study probably represent only the tip of the iceberg in the outbreak since mycobacterial culture is not routinely performed for diagnosis of TB in Haiti where GHESKIO laboratory is the only facility with culture capacity. For that reason the outbreak transmission chain could not be reconstructed and the index case was not identified.

Recent WGS studies estimated the evolution rate in settings with low TB and HIV prevalence as 0.3–0.5 SNPs per genome per year resulting in accumulation of maximum 5 genetic changes in three years and 10 genetic changes in 10 years [45, 46]. Based on the time of the earliest microbiologically diagnosed case, we estimate that the outbreak started in or before 2007. Individual isolates harbored 2 to 8 sequence variants not associated with drug resistance. Isolates with >5 SNPs came from patients with history of treatment with anti-tuberculosis drugs. Multiple treatment episodes like in the case of patient D create a “bottleneck” effect—killing part of the bacteria and selecting those with mutations providing any degree of survival advantage [44]. Overall the low number of SNPs found in our cases is in agreement with the estimate for molecular clock obtained in developed countries and is consistent with long periods of latent infection and few steps in the infection transmission chain in the L511P cluster.

To our knowledge this is the first report that delineates the fate of infections involving MTB strains with RIF MIC of 0.125 μg/ml, which is eight times lower than the currently accepted critical concentration of the drug. WGS confirmed that at least 7 out of the 8 cases in L511P cluster resulted from the clonal expansion of the same ancestral strain. Although the strain fitness was not determined in this study, it did not prevent transmission from mother to child, among distant family members, and between the casual social contacts. Immuno-compromised patient status was not a prerequisite for transmission since six of the eight patients who provided MTB isolates were HIV-negative.

Under the selective pressure of treatment with first line TB drugs, the strain initially resistant only to INH and to the very low level of RIF, acquired *embB* mutation at position M306. “Canonical” mutations in M306 codon of *embB* are necessary but not sufficient for development of a high-level resistance to EMB [47, 48]. However they are strongly associated with emergence of resistance to RIF and to other anti-tuberculosis drugs by altering the properties of the efflux pump and effectively lowering their concentration inside the cells [49]. Accordingly, M306I *embB* mutation precluded the acquisition of a secondary *rpoB* mutation in two independent instances. Both times RIF resistance level escalated from the baseline 0.125 μg/ml—to intermediate level of 0.5 μg/ml in patient A (L511P & M515T) and to a high resistance level of >8 μg/ml in patient C (L511P & D516C).

In conclusion, this study reports the first results obtained from a project to genotype MDR-TB isolates in Haiti with WGS. While only a very limited number of strains were sequenced, we already gained novel insights into the public health significance of MTB strains with a sub-critical resistance to RIF by demonstrating their transmissibility and potential for escalation of resistance level under conditions of treatment with RIF-based regimens. We expect that introduction of routine WGS of MDR-TB strains in Haiti will provide necessary data to improve diagnosis, deepen understanding about genetic determinants of resistance and help shaping more effective public health policies to combat the disease.

## Supporting Information

S1 Table L511P cluster—mutation found in genes linked to resistance to drugs and conventional DST results. List of drugs and genes adopted from [35]. Drugs with conventional susceptibility result are shown in bold.
NT—not tested
* One of eight isolates with double mutations L511P&D516C was resistant to RIF
** Position in *E*. *coli* genome shown in parentheses
(DOCX)

## References

[1] Gandhi NR, Nunn P, Dheda K, Schaaf HS, Zignol M, van Soolingen D, et al Multidrug-resistant and extensively drug-resistant tuberculosis: a threat to global control of tuberculosis. Lancet. 2010; 375: 1830–1843. 10.1016/S0140-6736(10)60410-2 20488523

[2] World Health Organization. Guidelines for surveillance of drug resistance in tuberculosis. Document WHO/TB/94.178. 1994; Available: http://who.int/tb/publications/1994/en/index.html.

[3] Traore H, Fissette K, Bastian I, Devleeschouwer M, Portaels F. Detection of rifampicin resistance in Mycobacterium tuberculosis isolates from diverse countries by a commercial line probe assay as an initial indicator of multidrug resistance. Int J Tuberc Lung Dis. 2000; 4(5): 481–484. 10815743

[4] World Health Organization. Companion handbook to the WHO guidelines for the programmatic management of drug-resistant tuberculosis. Document WHO/HTM/TB/2014. 2014; Available: http://www.who.int/tb/publications/pmdt_companionhandbook/en/ 25320836

[5] National Committee on Clinical Laboratory Standards. Susceptibility testing of Mycobacteria, Nocardiae, and other aerobic Actinomycetes Approved standard. NCCLS document M24-A. Wayne, Pennsylvania: NCCLS; 2003. 31339680

[6] Ramaswamy S, Musser JM. Molecular genetic basis of antimicrobial agent resistance in *Mycobacterium tuberculosis*: 1998 update. Tuber Lung Dis. 1998; 79(1): 3–29. 10645439 10.1054/tuld.1998.0002

[7] Telenti A, Imboden P, Marchesi F, Lowrie D, Cole S, Colston MJ, et al Detection of rifampicin-resistance mutations in *Mycobacterium tuberculosis* . Lancet. 1993; 341: 647–650. 8095569 10.1016/0140-6736(93)90417-f

[8] Boehme CC, Nabeta P, Hillemann D, Nicol MP, Shenai S, Krapp F, et al Rapid molecular detection of tuberculosis and rifampin resistance. N Engl J Med. 2010; 363(11): 1005–1015. 10.1056/NEJMoa0907847 20825313 PMC2947799

[9] Lawn SD, Brooks SV, Kranzer K, Nicol MP, Whitelaw A, Vogt M, et al Screening for HIV-associated tuberculosis and rifampicin resistance before antiretroviral therapy using the Xpert MTB/RIF assay: a prospective study. PLoS Med. 2011; 8(7): e1001067 10.1371/journal.pmed.1001067 21818180 PMC3144215

[10] Traore H, Fissette K, Bastian I, Devleeschouwer M, Portaels F. Detection of rifampicin resistance in Mycobacterium tuberculosis isolates from diverse countries by a commercial line probe assay as an initial indicator of multidrug resistance. Int J Tuberc Lung Dis. 2010; 4(5): 481–484. 10815743

[11] Van Deun A, Aung KJ, Bola V, Lebeke R, Hossain MA, de Rijk WB, et al Rifampin drug resistance tests for tuberculosis: challenging the gold standard. J Clin Microbiol. 2013; 51(8): 2633–2640. 10.1128/JCM.00553-13 23761144 PMC3719626

[12] Chen L, Gan X, Li N, Wang J, Li K, Zhang H. rpoB gene mutation profile in rifampicin-resistant Mycobacterium tuberculosis clinical isolates from Guizhou, one of the highest incidence rate regions in China. J Antimicrob Chemother. 2010; 65(6): 1299–1301. 10.1093/jac/dkq102 20356906

[13] Yip CW1, Leung KL, Wong D, Cheung DT, Chu MY, Tang HS, et al Denaturing HPLC for high-throughput screening of rifampicin-resistant Mycobacterium tuberculosis isolates. Int J Tuberc Lung Dis. 2006; 10(6): 625–630. 16776449

[14] Ocheretina O, Escuyer VE, Mabou MM, Royal-Mardi G, Collins S, Vilbrun SC, et al (2014) Correlation between Genotypic and Phenotypic Testing for Resistance to Rifampin in Mycobacterium tuberculosis Clinical Isolates in Haiti: Investigation of Cases with Discrepant Susceptibility Results. PLoS One. 2014; 9(3): e90569 10.1371/journal.pone.0090569 24599230 PMC3944071

[15] Van Deun A, Barrera L, Bastian I, Fattorini L, Hoffmann H, Kam KM, et al *Mycobacterium tuberculosis* strains with highly discordant rifampin susceptibility test results. J Clin Microbiol. 2009; 47(11): 3501–3506. 10.1128/JCM.01209-09 19759221 PMC2772627

[16] Jamieson FB, Guthrie JL, Neemuchwala A, Lastovetska O, Melano RG, Mehaffy C. Profiling of rpoB mutations and MICs for rifampin and rifabutin in Mycobacterium tuberculosis. J Clin Microbiol. 2014; 52(6): 2157–2162. 10.1128/JCM.00691-14 24740074 PMC4042728

[17] Williamson DA, Roberts SA, Bower JE, Vaughan R, Newton S, Lowe O, et al Clinical failures associated with *rpoB* mutations in phenotypically occult multidrug-resistant *Mycobacterium tuberculosis* . Int J Tuberc Lung Dis. 2011; 16(2): 216–220. 10.5588/ijtld.11.0178 22137551

[18] Ioerger TR1, Koo S, No EG, Chen X, Larsen MH, Jacobs WR Jr, et al, Genome analysis of multi- and extensively-drug-resistant tuberculosis from KwaZulu-Natal, South Africa. PLoS One. 2009; 4(11): e7778 10.1371/journal.pone.0007778 19890396 PMC2767505

[19] Billington OJ, McHugh TD, Gillespie SH. Physiological cost of rifampin resistance induced in vitro in Mycobacterium tuberculosis. Antimicrob Agents Chemother. 1999; 43(8): 1866–1869. 10428904 10.1128/aac.43.8.1866 PMC89382

[20] Mariam DH, Mengistu Y, Hoffner SE, Andersson DI. Effect of rpoB mutations conferring rifampin resistance on fitness of Mycobacterium tuberculosis. Antimicrob Agents Chemother. 2004; 48(4): 1289–1294. 15047531 10.1128/AAC.48.4.1289-1294.2004 PMC375340

[21] Gagneux S, Long CD, Small PM, Van T, Schoolnik GK, Bohannan BJ. The competitive cost of antibiotic resistance in Mycobacterium tuberculosis. Science. 2006; 312(5782): 1944–1946. 16809538 10.1126/science.1124410

[22] Bergval I, Kwok B, Schuitema A, Kremer K, van Soolingen D, et al Pre-existing isoniazid resistance but not the genotype of Mycobacterium tuberculosis drives rifampicin resistance codon preference in vitro. PLoS One. 2012; 7(1): e29108 10.1371/journal.pone.0029108 22235262 PMC3250395

[23] Blumberg H. M. Burman WJ, Chaisson RE, Daley CL, Etkind SC, Friedman LN, et al American Thoracic Society/Centers for Disease Control and Prevention/Infectious Diseases Society of America: treatment of tuberculosis. Am J Respir Crit Care Med. 2003 167(4), 603–662. 12588714 10.1164/rccm.167.4.603

[24] World Health Organization & Stop TB Initiative. Treatment of tuberculosis: guidelines. 4th edn, Document WHO/HTM/TB/2009.420. 2009; Available: http://whqlibdoc.who.int/publications/2010/9789241547833_eng.pdf

[25] World Health Organization. Guidelines for the programmatic management of drug-resistant tuberculosis. Document WHO/HTM/TB/2008.402. 2008; Available: http://whqlibdoc.who.int/publications/2008/9789241547581_eng.pdf?ua=1

[26] World Health Organization. Treatment of tuberculosis: guidelines for national programmes- 3rd ed. Document WHO/CDS/TB/2003.313. 2003; Available: http://whqlibdoc.who.int/publications/2010/9789241547833_eng.pdf

[27] Gardy JL, Johnston JC, Ho Sui SJ, Cook VJ, Shah L, Brodkin E, et al, Whole-genome sequencing and social-network analysis of a tuberculosis outbreak. N Engl J Med. 2011; 364(8): 730–739. 10.1056/NEJMoa1003176 21345102

[28] van Soolingen D, Hermans PW, de Haas PE, Soll DR, van Embden JD. Occurrence and stability of insertion sequences in Mycobacterium tuberculosis complex strains: evaluation of an insertion sequence-dependent DNA polymorphism as a tool in the epidemiology of tuberculosis. J Clin Microbiol. 1991; 29(11): 2578–2586. 1685494 10.1128/jcm.29.11.2578-2586.1991 PMC270376

[29] McKenna A, Hanna M, Banks E, Sivachenko A, Cibulskis K, Kernytsky A, et al The Genome Analysis Toolkit: a MapReduce framework for analyzing next-generation DNA sequencing data. *Genome Res*. 2010; 20: 1297–1303. 10.1101/gr.107524.110 20644199 PMC2928508

[30] DePristo M, Banks E, Poplin R, Garimella K, Maguire J, Hartl C, et al A framework for variation discovery and genotyping using next-generation DNA sequencing data. *Nature Genetics*. 2011; 43: 491–498. 10.1038/ng.806 21478889 PMC3083463

[31] McLaren W, Pritchard B, Rios D, Chen Y, Flicek P, Cunningham F. Deriving the consequences of genomic variants with the Ensembl API and SNP Effect Predictor. *BMC Bioinformatics*. 2010; 26(16): 2069–2070. 10.1093/bioinformatics/btq330 20562413 PMC2916720

[32] Ocheretina O, Merveille YM, Mabou MM, Escuyer VE, Dunbar SA, Johnson WD, et al Use of Luminex MagPlex(R) Magnetic Microspheres for High-Throughput Spoligotyping of *M*. *tuberculosis* Isolates in Port-au-Prince, Haiti. J Clin Microbiol. 2013; 51(7): 2232–2237. 10.1128/JCM.00268-13 23658258 PMC3697689

[33] Köser CU, Bryant JM, Becq J, Török ME, Ellington MJ, Marti-Renom MA, et al Whole-genome sequencing for rapid susceptibility testing of M. tuberculosis. N Engl J Med. 2013; 369(3): 290–292. 10.1056/NEJMc1215305 23863072 PMC3836233

[34] Zhang H, Li D, Zhao L, Fleming J, Lin N, Wang T, et al Genome sequencing of 161 Mycobacterium tuberculosis isolates from China identifies genes and intergenic regions associated with drug resistance. Nat Genet. 2013; 45(10): 1255–1260. 10.1038/ng.2735 23995137

[35] Casali N, Nikolayevskyy V, Balabanova Y, Harris SR, Ignatyeva O, Kontsevaya I, et al Evolution and transmission of drug-resistant tuberculosis in a Russian population. Nat Genet. 2014; 46(3): 279–286. 10.1038/ng.2878 24464101 PMC3939361

[36] Comas I, Borrell S, Roetzer A, Rose G, Malla B, Kato-Maeda M, et al Whole-genome sequencing of rifampicin-resistant *Mycobacterium tuberculosis* strains identifies compensatory mutations in RNA polymerase genes. Nat Genet. 2011; 44(1): 106–110. 10.1038/ng.1038 22179134 PMC3246538

[37] van Belkum A, Struelens M, de Visser A, Verbrugh H, Tibayrenc M. Role of genomic typing in taxonomy, evolutionary genetics, and microbial epidemiology. Clin Microbiol Rev. 2001; 14(3): 547–560. 11432813 10.1128/CMR.14.3.547-560.2001 PMC88989

[38] Andrews JR, Morrow C, Wood R. Modeling the role of public transportation in sustaining tuberculosis transmission in South Africa. Am J Epidemiol. 2013; 177(6): 556–561. 10.1093/aje/kws331 23423215 PMC3657527

[39] Andrews JR, Morrow C, Walensky RP, Wood R. Integrating social contact and environmental data in evaluating tuberculosis transmission in a South African township. J Infect Dis. 2014; 210(4): 597–603. 10.1093/infdis/jiu138 24610874 PMC4133578

[40] van der Spuy GD, Warren RM, van Helden PD. The role of molecular epidemiology in low-income, high-burden countries. Int J Tuberc Lung Dis. 2009; 13(4): 419–420. 19335944

[41] International Organization for Migration, Displacement Tracking Matrix, Haiti. 2013; Available: https://www.iom.int/files/live/sites/iom/files/pbn/docs/DTM_V2_Report_March-31_2013_English-final.pdf

[42] Kremer K, Arnold C, Cataldi A, Gutiérrez MC, Haas WH, Panaiotov S, et al Discriminatory power and reproducibility of novel DNA typing methods for Mycobacterium tuberculosis complex strains. J Clin Microbiol. 2005; 43(11): 5628–5638. 16272496 10.1128/JCM.43.11.5628-5638.2005 PMC1287774

[43] Kremer K, van Soolingen D, Frothingham R, Haas WH, Hermans PW, Martín C, et al Comparison of methods based on different molecular epidemiological markers for typing of Mycobacterium tuberculosis complex strains: interlaboratory study of discriminatory power and reproducibility. J Clin Microbiol. 1999; 37(8): 2607–2618. 10405410 10.1128/jcm.37.8.2607-2618.1999 PMC85295

[44] Kato-Maeda M, Ho C, Passarelli B, Banaei N, Grinsdale J, Flores L, et al Use of whole genome sequencing to determine the microevolution of Mycobacterium tuberculosis during an outbreak. PLoS One. 2013; 8(3): e58235 10.1371/journal.pone.0058235 23472164 PMC3589338

[45] Walker TM, Ip CL, Harrell RH, Evans JT, Kapatai G, Dedicoat MJ, et al, Whole-genome sequencing to delineate Mycobacterium tuberculosis outbreaks: a retrospective observational study. Lancet Infect Dis. 2013; 13(2): 137–146. 10.1016/S1473-3099(12)70277-3 23158499 PMC3556524

[46] Bryant JM, Schürch AC, van Deutekom H, Harris SR, de Beer JL, de Jager V, et al Inferring patient to patient transmission of Mycobacterium tuberculosis from whole genome sequencing data. BMC Infect Dis. 2013; 13:110 10.1186/1471-2334-13-110 23446317 PMC3599118

[47] Safi H, Sayers B, Hazbón MH, Alland D. Transfer of embB codon 306 mutations into clinical Mycobacterium tuberculosis strains alters susceptibility to ethambutol, isoniazid, and rifampin. Antimicrob Agents Chemother. 2008; 52(6): 2027–2034. 10.1128/AAC.01486-07 18378710 PMC2415778

[48] Safi H, Lingaraju S, Amin A, Kim S, Jones M, Holmes M, et al Evolution of high-level ethambutol-resistant tuberculosis through interacting mutations in decaprenylphosphoryl-β-D-arabinose biosynthetic and utilization pathway genes. Nat Genet. 2013; 45(10): 1190–1197. 10.1038/ng.2743 23995136 PMC6103293

[49] Srivastava S, Musuka S, Sherman C, Meek C, Leff R, Gumbo T. Efflux-pump-derived multiple drug resistance to ethambutol monotherapy in Mycobacterium tuberculosis and the pharmacokinetics and pharmacodynamics of ethambutol. J Infect Dis. 2010; 201(8): 1225–31. 10.1086/651377 20210628 PMC2838947

