# Supplementary material for: Whole Genome Sequencing Investigation of a Tuberculosis Outbreak in Port-au-Prince, Haiti Caused by a Strain with a “Low-Level” rpoB Mutation L511P – Insights into a Mechanism of Resistance Escalation
Source: PLoS One. 2015 Jun 3;10(6):e0129207. doi: 10.1371/journal.pone.0129207 (PMC4454571; doi:10.1371/journal.pone.0129207)
Supplement: S1 Table — List of drugs and genes adopted from [35]. Drugs with conventional susceptibility result are shown in bold. NT—not tested * One of eight isolates with double mutations L511P&D516C was resistant to RIF ** Position in E. coli genome shown in parentheses (DOCX) [file pone.0129207.s001.docx]

**S1 Table. L511P cluster - mutation found in genes linked to resistance to drugs and conventional DST results.**

| **Antibiotic** | **Phenotype** | **Known  resistance  gene(s)** | **Position in protein** | **AA change** | **Codon change** |
| --- | --- | --- | --- | --- | --- |
| **Amikacin,** | S | rpsL (Rv0682) | 121 | K | aaA/aaG |
| **Capreomycin,** | S | tap (Rv1258c) |  |  |  |
| **Kanamycin,** | S | rrs (MTB000019) |  |  |  |
| **Streptomycin,** | S | rrl (MTB000020) |  |  |  |
| Viomycin | NT | tlyA (Rv1694) | 11 | L | ctA/ctG |
|  |  | eis (Rv2416c) |  |  |  |
|  |  | whiB7 (whmC, Rv3197A) |  |  |  |
|  |  | Rv3728 |  |  |  |
|  |  | gid (gidB, Rv3919c) |  |  |  |
|  |  |  |  |  |  |
| AU1235, BM212, C215, SQ109 | NT | mmpL3 (Rv0206c) |  |  |  |
|  |  |  |  |  |  |
| AZD5847, Linezolid, Sutezolid (PNU-100480) | NT | rplC (Rv0701) |  |  |  |
|  |  | rrl (MTB000020) |  |  |  |
|  |  |  |  |  |  |
| Bedaquiline (Sirturo, TMC -207, R207910) | NT | atpE (Rv1305) |  |  |  |
|  |  |  |  |  |  |
| BTZ043, DNB1, VI-9376, 377790 | NT | dprE1 (Rv3790) |  |  |  |
|  |  |  |  |  |  |
| Clarithromycin | NT | ermMT (erm37, Rv1988) |  |  |  |
|  |  | whiB7 (whmC, Rv3197A) |  |  |  |
|  |  |  |  |  |  |
| Clofazimine, Thioridazine | NT | ndh (Rv1854c) |  |  |  |
|  |  |  |  |  |  |
| **Cycloserine** | S | cycA (Rv1704c) | 93 | R/L | cGg/cTg |
|  |  | ddlA (ddl, Rv2981c) |  |  |  |
|  |  | alr (Rv3423c) | 197 | L | ctG/ctC |
|  |  |  |  |  |  |
| Delamanid  (OPC‐67683), PA‐824 | NT | fgd1 (Rv0407) |  |  |  |
|  |  | fbiC (Rv1173) |  |  |  |
|  |  | fbiA (Rv3261) |  |  |  |
|  |  | fbiB (Rv3262) |  |  |  |
|  |  | ddn (Rv3547) |  |  |  |
|  |  |  |  |  |  |
| **Ethambutol** | S | embR (Rv1267c) |  |  |  |
|  |  | embC (Rv3793) | 927 | R | cgC/cgT |
|  |  | embA (Rv3794) |  |  |  |
|  |  | embB (Rv3795) | 306 | M/I | atG/atA |
|  |  |  |  |  |  |
| **Ofloxacin,** | S | gyrB (Rv0005) | 526 | K/Q | Aag/Cag |
| Gatifloxacin, Levofloxacin, Moxifloxacin, | NT | gyrA (Rv0006) | 21 | E/Q | Gag/Cag |
|  |  | mfpA (Rv3361c) |  |  |  |
|  |  |  |  |  |  |
| Imipenem, Meropenem/ co-amoxiclav | NT | pbpA (Rv0016c) |  |  |  |
|  |  | blaC (Rv2068c) |  |  |  |
|  |  | ldtA (ldtMt1, Rv0116c) |  |  |  |
|  |  | ldtB (ldtMt2, Rv2518c) |  |  |  |
|  |  | dacB2 (dacB, Rv2911) |  |  |  |
|  |  |  |  |  |  |
| **Isoniazid,** | R | mshA (Rv0486) |  |  |  |
| **Ethionamide,** | S | hadA (Rv0635) |  |  |  |
| Prothionamide, Thioacetazone | NT | adB (Rv0636) |  |  |  |
|  |  | hadC (Rv0637) |  |  |  |
|  |  | mmaA4 (Rv0642c) |  |  |  |
|  |  | mmaA3 (Rv0643c) |  |  |  |
|  |  | mmaA2 (Rv0644c) |  |  |  |
|  |  | mshB (Rv1170) |  |  |  |
|  |  | sigI (Rv1189) |  |  |  |
|  |  | fabG1 (mabA, Rv1483) |  |  |  |
|  |  | inhA (Rv1484) |  |  |  |
|  |  | ndh (Rv1854c) |  |  |  |
|  |  | katG (Rv1908c) | 315 | S/T | aGc/aCc |
|  |  | furA (Rv1909c) |  |  |  |
|  |  | mshC (cysS2, Rv2130) |  |  |  |
|  |  | ahpC (Rv2428) |  |  |  |
|  |  | nudC (Rv3199c) |  |  |  |
|  |  | nat (nhoA, Rv3566c) |  |  |  |
|  |  | ethA (aka, etaA, Rv3854c) |  |  |  |
|  |  | ethR (aka, etaR, Rv3855) |  |  |  |
|  |  |  |  |  |  |
| **Para‐aminosalicylic acid**, | S | folC  (Rv2447c) |  |  |  |
| Co-trimoxazole | NT | ribD  (Rv2671) |  |  |  |
|  |  | dfrA (Rv2763c) |  |  |  |
|  |  | thyA (Rv2764c) |  |  |  |
|  |  | folP1 (Rv3608c) |  |  |  |
|  |  |  |  |  |  |
| **Pyrazinamide** | S | rpsA (Rv1630) |  |  |  |
|  |  | pncA (Rv2043c) |  |  |  |
|  |  | panD (Rv3601c) |  |  |  |
|  |  |  |  |  |  |
| **Rifampicin,** | S* | rpoB (Rv0667) | 436 (511)** | L/P | cTg/cCg |
| **Rifabutin,** | S |  | 440 (515)** | M/T | aTg/aCg |
| Rifapentine | NT |  | 441 (516)** | D/C | GAc/TGc |
|  |  | rpoC (Rv0668) |  |  |  |
|  |  | rpoA (Rv3457c) |  |  |  |
